# Impact of prenatal marijuana exposure on adolescent brain structural and functional connectivity and behavioural outcomes

**DOI:** 10.1093/braincomms/fcae001

**Published:** 2024-01-08

**Authors:** Ramana V Vishnubhotla, Sidra T Ahmad, Yi Zhao, Rupa Radhakrishnan

**Affiliations:** Department of Radiology and Imaging Sciences, Indiana University School of Medicine, Indianapolis, IN 46202, USA; Indiana University School of Medicine, Indianapolis, IN 46202, USA; Department of Biostatistics and Health Data Science, Indiana University School of Medicine, Indianapolis, IN 46202, USA; Department of Radiology and Imaging Sciences, Indiana University School of Medicine, Indianapolis, IN 46202, USA

**Keywords:** ABCD, prenatal marijuana exposure, functional connectivity, structural connectivity, graph networks

## Abstract

There has been an increase in the number of women using marijuana whilst pregnant. Previous studies have shown that children with prenatal marijuana exposure have developmental deficits in memory and decreased attentiveness. In this study, we assess whether prenatal marijuana exposure is associated with alterations in brain regional morphometry and functional and structural connectivity in adolescents. We downloaded behavioural scores and subject image files from the Adolescent Brain Cognitive Development^SM^ Study. A total of 178 anatomical and diffusion magnetic resonance imaging files (88 prenatal marijuana exposure and 90 age- and gender-matched controls) and 152 resting-state functional magnetic resonance imaging files (76 prenatal marijuana exposure and 76 controls) were obtained. Behavioural metrics based on the parent-reported child behavioural checklist were also obtained for each subject. The associations of prenatal marijuana exposure with 17 subscales of the child behavioural checklist were calculated. We assessed differences in brain morphometry based on voxel-based and surface-based morphometry in adolescents with prenatal marijuana exposure versus controls. We also evaluated group differences in structural and functional connectivity in adolescents for region-to-region connectivity and graph theoretical metrics. Interactions of prenatal marijuana exposure and graph networks were assessed for impact on behavioural scores. Multiple comparison correction was performed as appropriate. Adolescents with prenatal marijuana exposure had greater abnormal or borderline child behavioural checklist scores in 9 out of 17 subscales. There were no significant differences in voxel- or surface-based morphometry, structural connectivity or functional connectivity between prenatal marijuana exposure and controls. However, there were significant differences in prenatal marijuana exposure–graph network interactions with respect to behavioural scores. There were three structural prenatal marijuana exposure–graph network interactions and seven functional prenatal marijuana exposure–graph network interactions that were significantly associated with behavioural scores. Whilst this study was not able to confirm anatomical or functional differences between prenatal marijuana exposure and unexposed pre-adolescent children, there were prenatal marijuana exposure–brain structural and functional graph network interactions that were significantly associated with behavioural scores. This suggests that altered brain networks may underlie behavioural outcomes in adolescents with prenatal marijuana exposure. More work needs to be conducted to better understand the prognostic value of brain structural and functional network measures in prenatal marijuana exposure.

## Teaser

Vishnubhotla *et al*. demonstrate that prenatal marijuana exposure is associated with worse behavioural scores in adolescents. Prenatal marijuana exposure also impacts the association between several structural and functional graph network measures and behavioural scores, suggesting that altered brain networks could underlie adverse behavioural outcomes in adolescents with prenatal marijuana exposure.

## Graphical Abstract

**Graphical Abstract:**
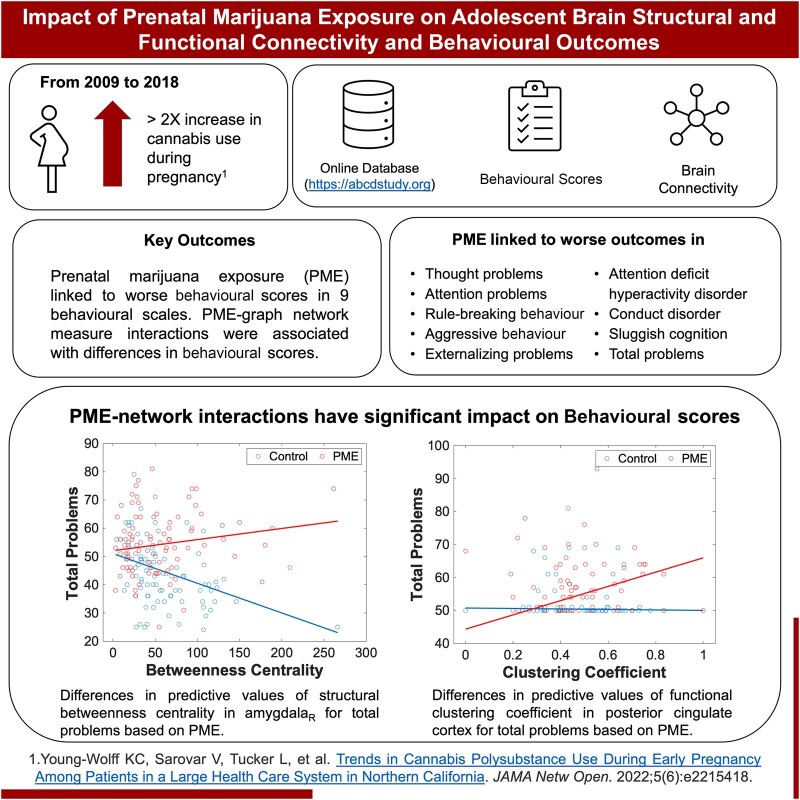

## Introduction

Several studies have shown an increasing prevalence of marijuana use following legalization trends, and the United Nations Office on Drugs and Crime reported marijuana as the most commonly used illicit drug globally in 2016.^1^ Along with increasing rates of marijuana use, there has been an increase in the number of women using marijuana whilst pregnant. Alleviation of nausea, vomiting and morning sickness have been cited as common reasons for marijuana use during pregnancy. Additionally, there is an increasing perception of safety with prenatal marijuana exposure (PME), as many pregnant women report being unaware of any adverse effects associated with PME.^2-4^ Marijuana is the most common recreational drug used in pregnancy.^5^ A cross-sectional study found that out of 367,403 pregnancies between 2009 and 2017 in the Kaiser Permanente Northern California population, self-reported daily cannabis use during pregnancy was 3.38% in 2017.^3^ In a follow-up study, cannabis-only use during pregnancy rose to 6.3% in 2018.^6^ The 2020 National Survey of Drug Use and Health reported marijuana use in the past month among pregnant women aged 15–44 as 8%.^7^ The National Institute on Drug Abuse estimates that rates may be higher since women were twice as likely to screen positive for marijuana use in a urine test than self-report.^8^ Therefore, these are likely underestimates because data from most studies is self-reported.

Despite the increasing prevalence of PME, there is a relative lack of knowledge on the long-term effects of PME. Previous studies have shown that PME leads to developmental deficits—such as gaps in problem-solving skills and memory, increased depressive and anxiety symptoms, and decreased ability to remain attentive in school-aged children with PME compared with controls.^9-12^ The Ottawa Prenatal Prospective Study^13,14^ showed developmental challenges in children with PME that persisted from birth until 12 years of age. These developmental challenges included decreased verbal memory, poorer sustained attention, increased risk for hyperactivity and impulsivity and poorer executive functioning. Other researchers have shown that these cognitive and behavioural challenges continued onto adulthood as those with PME showed decreased response inhibition, and significantly greater brain activity compared with unexposed controls when performing executive functioning tasks.^15^

Effects of PME on the brain have been assessed through neuroimaging studies. Functional magnetic resonance imaging (fMRI) studies reveal impaired executive functioning,^15^ response inhibition^16^ and visuospatial working memory^17^ in children with PME compared with controls. Additionally, prenatal drug exposure, including marijuana, has shown to impact brain functional network connectivity.^18-21^ Assessing brain network connectivity could help predict those at risk for developmental conditions. For example, children with attention-deficit hyperactivity disorder (ADHD) are shown to have altered brain network connectivity,^22-24^ and risk of behavioural problems in children with ADHD children are associated with altered functional connectivity.^25^ Assessing structural connectivity is also important as white matter microstructural alterations have been associated with disruptive behaviours.^26^

Brain structure and function can be assessed through direct connections between regions of interest (ROIs) and interactions between regions or through organized frameworks called graph networks. Graph networks are relationships or connections between multiple brain regions that are mathematically assessed utilizing systems composed of nodes (specific brain region) and edges (connections between nodes). Several metrics are used to understand how inter-connected these nodes are, that may be global, comprising the entire network, or local, in a particular region of the network. These graph network metrics provide information on global and local network connectivity that cannot be assessed by simple ROI-to-ROI connectivity, which assesses each connection independently.

Graph theory metrics have been applied in neuroimaging to understand networks in the human brain.^27-31^ For example, individuals with ADHD had lower global and local efficiencies (measures of how well a network exchanges information), along with a longer characteristic path length (indicating lower efficiency).^32^ In the adult population, individuals with ADHD had lower local efficiencies and altered clustering coefficients (a measure of the extent regions cluster together) in multiple regions.^33^ Additionally, reduced values in another graph network measure, betweenness centrality (a measure of the amount of influence a region has in the flow of information), in the hippocampus and prefrontal cortex were associated with greater stress.^34^

Since brain structural and functional connectivity could be an important measure of developmental and behavioural problems and PME can be associated with adverse behavioural and developmental outcomes, we assess (i) the impact of PME on developmental and behavioural problems, and (ii) the impact of PME on the associations of altered brain morphology, structural connectivity and functional connectivity with behavioural outcomes in adolescents.

## Methods

### Gathering data

Data were acquired from the Adolescent Brain Cognitive Development^SM^ (ABCD®) Study (https://abcdstudy.org), held in the NIMH Data Archive.^35,36^ Subjects were screened for PME, excluding exposure to substances such as alcohol, tobacco and other illicit drugs. Controls were screened and excluded for exposure to marijuana, alcohol, tobacco and other illicit drugs. Additionally, controls were selected to have a similar gender composition to the PME subjects. Demographic characteristics, such as age, sex, mean age, mean birth weight, maternal prescription medications, premature birth and maternal education, were compared between the PME and control groups. The parent-reported child behavioural checklist (CBCL)^37^ scores were also downloaded for each subject.

Minimally processed resting-state fMRI (rs-fMRI) images, diffusion-weighted images and T_1_-weighted anatomical images were also downloaded. Image samples were processed as described by Hagler *et al*.^38^ Briefly, fMRI data had B0 correction, unwarping and motion correction. Diffusion images had B0 correction, unwarping, motion correction and eddy current correction. Anatomical images had unwarping, intensity inhomogeneity correction and rigid body registration.

### Behavioural metrics

Behavioural metrics were based on a parent-reported CBCL^37^ and used 17 subscales. These include anxiety/depression, withdrawn/depression, somatic complaints, social problems, thought problems, attention problems, rule-breaking behaviour, aggressive behaviour, internalizing problems, externalizing problems, ADHD, oppositional disorder, conduct disorder, sluggish cognition, obsessive-compulsive disorder, stress problems and total problems. Internalizing problems included scales for anxiety, withdrawal, depression and somatic complaints whilst externalizing problems included scales for rule-breaking and aggressive behaviours.^39^ There were specific scales for the Diagnostic and Statistical Manual of Mental Disorders, Fourth Edition criteria including ADHD, oppositional and conduct disorders.^40^ The scales used were normative, where 50 was considered average and clinical cut-offs of 65–69 for borderline and 70+ for clinically abnormal values. The impact of demographic characteristics such as age, sex, presence of PME, maternal education and partner education was assessed. Differences in CBCL clinical categories between PME and control groups were also assessed.

### Voxel-based and surface-based morphometry

Voxel-based morphometry and surface-based morphometry were calculated from T_1_-weighted anatomical images using Computational Anatomy Toolbox 12.6 (CAT12) (Jena, Germany),^41^ a toolbox for Statistical Parametric Mapping, version 12 (SPM12).^42^ Steps for voxel-based morphometry processing include denoising,^43^ affine pre-processing, local adaptive segmentation, skull stripping and spatial normalization.^44^ Voxel-based morphometry grey matter segmentations were then smoothed at 8-mm full width at half maximum. Additional steps for surface-based morphometry processing utilize the projection-based thickness method^45^ along with topology correction and spherical mapping.^46^ Surface data were resampled based on a 32-K vertices surface mesh^47^ and smoothed to 15-mm full width at half maximum. For statistical analysis, two contrasts were set—PME > control and control > PME. Clusters less than 10 voxels were excluded. Total intracranial volume was used as a covariate for voxel-based morphometry.

### Tract-based spatial statistics

Diffusion-weighted images, b-value, and b-vector files were downloaded from the ABCD® Study database. Tract-based spatial statistics^48^ were performed using the FMRIB (for fMRI of the brain) software library (FSL, Oxford, UK).^49^ First, fractional anisotropy (FA) images were created by fitting a tensor model using FMRIB’s diffusion toolbox and then brain extracted using FSL’s brain extraction tool.^50^ Subjects’ data were aligned into a common space using FMRIB’s non-linear image registration tool^51,52^ using a b-spline representation of the registration warp field.^53^ A mean FA skeleton was generated representing the centre of group tracts. Each subject’s FA data was projected onto the skeleton and voxelwise cross-subject statistics were generated. Data for mean diffusivity and axial diffusivity were obtained and data for radial diffusivity were calculated by taking the mean of the second and third eigenvalues. Mean diffusivity, axial diffusivity and radial diffusivity data were projected onto mean FA skeleton and cross-subject statistics were generated similar to FA. Comparisons were made between PME and unexposed controls using a general linear model and voxelwise statistics were performed using threshold-free cluster enhancement in *randomize.*^54^

### Tractography

Diffusion-weighted images, b-value, and b-vector files were downloaded from the ABCD® Study database. The b-tables were imported and corrected using DSI Studio (http://dsi-studio.labsolver.org) using a population average template.^55^ Diffusion data were reconstructed in the Montreal Neurological Institute space using q-space diffeomorphic reconstruction^56^ and aligned with the International Consortium for Brain Mapping template.^57,58^ Tractography was performed on the whole brain with DSI Studio using a deterministic fibre tracking algorithm^59^ with a diffusion sampling ratio of 1.25. Three million tracts were calculated for each subject. The quantitative anisotropy threshold was set to software optimization. The angular threshold was set at 45° and the step size was 0.75 mm. Track lengths shorter than 20 mm or longer than 200 mm were discarded. The International Consortium for Brain Mapping template^57,58^ was registered to subject space through non-linear transformation. Brain parcellation regions were based on the automated anatomical labelling version 2 atlas.^60^ Connectivity matrices and graph network measures were calculated in DSI Studio based on fibre count.

### Structural pairwise connectivity

Connectivity matrices with 120 ROIs were collected for each subject based on fibre count. Regions involving the cerebellum and vermis were excluded, leaving 94 ROIs for the analysis. The count data were firstly normalized via square root transformation. Robust linear regression was employed to assess the significance of PME on connectivity, where predictors include age, sex, PME, maternal education and partner education.

### Structural graph network measures

Network measures for fibre count were collected for each subject within DSI Studio based on the Brain Connectivity Toolbox.^61^ Prior studies have shown altered graph networks in developmental conditions.^32-34^ Therefore, we evaluated network measures including average path length, global efficiency, local efficiency, betweenness centrality and clustering coefficient. Networks were based on weighted values. The significance of PME on network measures was assessed using a linear regression. Age, sex, PME, maternal education and partner education were predictors and network measures were the response variables.

### rs-fMRI pre-processing

Pre-processing and analysis of rs-fMRI data were performed with CONN Toolbox (Cambridge, MA).^62,63^ Samples obtained from the ABCD database were already minimally processed with B0 correction, unwarping and motion correction as described by Hagler *et al*.^38^ that we used for further analysis. Functional and structural MRI data were normalized to the standard Montreal Neurological Institute T_1_ template using a direct normalization process. Data were segmented into grey matter, white matter and cerebrospinal fluid.^64,65^ Isotropic resolution of 1 mm for structural images and 2 mm for functional images were used. Next, the data were smoothed using spatial convolution with a Gaussian kernel of 8-mm full width at half maximum.^63^

Denoising involved removing of noise from white matter and cerebrospinal fluid,^66,67^ scrubbing^68^ and session effects. For temporal band pass filtering, the lower frequency threshold was 0.008 Hz, and the upper frequency threshold was 0.09 Hz. Filtering was performed after regression to avoid mismatch in nuisance regressor procedure.^63,69^

### Independent component analysis

Independent component analysis was conducted using CONN Toolbox^62^ based on the methodology as described by Calhoun *et al*.^70^ Comparisons were made between PME and unexposed controls. Clusters less than five voxels in size were discarded.

### rs-fMRI analysis

Functional connectivity was assessed between ROIs using CONN Toolbox.^62^ ROI-to-ROI analysis was performed based on 106 regions of the Harvard–Oxford atlas,^71-74^ distributed by Conn Toolbox. Cerebellar regions were excluded from the analysis. Statistical analysis was also performed in CONN Toolbox. Comparisons were made between PME and unexposed controls using a weighted general linear model.^75^ Analyses were conducted on Fisher *z*-transformed correlations.

### Functional graph network measures

Functional graph network measures were obtained using CONN Toolbox based on the Brain Connectivity Toolbox.^61^ Prior studies have shown altered graph networks in developmental conditions.^32-34^ Therefore, we evaluated network measures including average path length, global efficiency, local efficiency, betweenness centrality and clustering coefficient. The significance of PME on network measures was assessed using a robust linear regression. Age, sex, PME, maternal education and partner education were the predictors and network measures were the response variables.

### Correlating behavioural data with graph network measures

Clinically relevant CBCL metrics were correlated with graph network measures and PME to assess their impact on clinical scores. The significance of network measures and PME on clinical scores was assessed using linear regression for structural and functional data. Age, sex, PME, maternal education, partner education and network measure were the predictors and CBCL scores were the response variables. An interaction between PME and network measures was included in the analysis so that significance was assessed for each group separately, as well as for the interaction term.

### Statistical analysis

Statistical analyses were performed using R Studio (posit.co/products/open-source/rstudio/), MATLAB® (mathworks.com/products/matlab.html; MathWorks; Natick, MA, USA), and within individual processing packages.

The impact of demographic characteristics such as age, sex, presence of PME, maternal education and partner education on behavioural measures was assessed using a general linear model. Differences in clinical categories were calculated using a Fisher’s exact test. Multiplicity was corrected following the Benjamini–Hochberg procedure to control for the false discovery rate.^76^ An adjusted *P*-value of <0.05 was considered significant.

Statistics for voxel-based and surface-based morphometry were calculated in SPM, statistics for TBBS were calculated in FSL, and statistics for fMRI independent component analysis were calculated in CONN Toolbox. Structural ROI-to-ROI connectivity was calculated using the sum of square errors followed by a robust linear regression. Functional ROI-to-ROI analysis was conducted in CONN Toolbox. Structural and functional graph network measures were calculated in DSI Studio and CONN Toolbox, respectively. Statistical comparisons of graph network measures were performed using linear regressions. Multiplicity was corrected following the Benjamini–Hochberg procedure to control for the false discovery rate.^76^ In all cases, significance was determined by an adjusted *P*-value of <0.05.

## Results

### Demographics

There were 178 subjects included in this study—88 (35 males) prenatally exposed to marijuana and 90 (37 males) without prenatal marijuana exposure. Both groups did not have history of prenatal exposure to other substances, such as alcohol, tobacco and other illicit drugs. Maternal education level was significantly different for PME versus unexposed controls (*P* = 0.0013). Factors such as birth weight, maternal prescription medications and premature birth were not statistically different. The demographic data are summarized in Table 1.

**Table 1:** Demographic information of children with PME and unexposed controls

	PME	Controls	*P*-value
Number	88	90	
Males	35	37	0.977^b^
Mean age (years) (SD)	9.9 (0.59)	9.9 (0.65)	0.879^a^
Mean birth weight (lbs.)	6.9	7.1	0.405^a^
Taking prescription medications	9	11	0.813^b^
Premature birth	15	13	0.684^b^
Maternal college degree	18	39	0.001^b,*^
Number (fMRI cohort)	76	76	
Males (fMRI cohort)	30	27	0.738^b^
Mean age of fMRI cohort (years) (SD)	9.9 (0.58)	9.9 (0.68)	0.694^a^

^a^Calculated by an unpaired *t*-test.

^b^Calculated using a Fisher’s exact test.

^*^Significant.

### Behavioural metrics

Behavioural metrics were based on the parent-reported CBCL^37^ from which we used data from 17 subscales. These scales had clinical cut-offs of 65–69 for borderline, and 70+ for clinical cases. Mean values were significantly greater in those with PME for all scales except the anxiety/depression and somatic complaint scales. Based on a general linear model, PME had a significant effect on 16 out of the 17 scales after correcting for multiple comparisons. No other variables were significant after multiple comparison corrections (Table 2). Clinical outcomes were significantly different in those with PME compared with unexposed controls in 9 of 17 scales. These include thought problems, attention problems, rule-breaking behaviour, aggressive behaviour, externalizing problems, ADHD, conduct disorder, sluggish cognition and total problems. The data are summarized in Table 3. Full data for this table are shown in Supplementary Table 1.

**Table 2:** A linear regression was performed with age, sex, maternal education, partner education and PME as predictors and behavioural scores as response variables

	*t*-stat	*P*-value	*P*-FDR
Anxiety/depression	2.04	0.043	0.046*
Withdrawn/depressed	2.53	0.012	0.014*
Somatic complaints	1.23	0.219	0.219
Social problems	3.69	<0.001	<0.001*
Thought problems	4.08	<0.001	<0.001*
Attention problems	4.43	<0.001	<0.001*
Rule breaking behaviour	3.74	<0.001	<0.001*
Aggressive behaviour	3.88	<0.001	<0.001*
Internalizing problems	3.10	0.002	0.003*
Externalizing problems	4.67	<0.001	<0.001*
ADHD	4.67	<0.001	<0.001*
Oppositional disorder	3.55	<0.001	<0.001*
Conduct disorder	3.64	<0.001	<0.001*
Sluggish cognitive	4.28	<0.001	<0.001*
Obsessive-compulsive disorder	2.79	0.006	0.007*
Stress problems	3.45	<0.001	<0.001*
Total problems	5.47	<0.001	<0.001*

All predictors except for PME were not significant after multiple comparison corrections. Behavioural measures for which PME was a significant predictor are shown in this table. A false discovery rate less than 0.05 was considered significant.

^*^Significant.

**Table 3:** CBCL clinical classifications based on sub-scale scores for PME and unexposed children

	*P*-value	*P*-FDR
Anxiety/depression	0.316	0.335
Withdrawn/depressed	0.185	0.225
Somatic complaints	0.230	0.261
Social problems	0.109	0.16
Thought problems	0.002	0.02^a^
Attention problems	0.006	0.025^a^
Rule breaking behaviour	0.021	0.042^a^
Aggressive behaviour	0.021	0.042^a^
Internalizing problems	0.113	0.16
Externalizing problems	0.007	0.025^a^
ADHD	0.002	0.02^a^
Oppositional disorder	0.034	0.058
Conduct disorder	0.022	0.042^a^
Sluggish cognitive	0.014	0.04^a^
Obsessive-compulsive disorder	0.180	0.225
Stress problems	0.371	0.371
Total problems	0.007	0.025^a^

Comparisons were calculated using a Fisher’s exact test and multiple comparison correction was based on false discovery rate. A false discovery rate less than 0.05 was considered significant.

^a^Significant.

### Voxel-based and surface-based morphometry

Brain anatomical measurements for volume and surface thickness were assessed using voxel-based and surface-based morphometry, respectively, for 88 PME and 90 unexposed children. There were no clusters or regions showing significant differences. The results are displayed in Supplementary Figs 1 and 2.

### Voxelwise tract-based spatial statistics

Tract-based spatial statistics were performed for diffusion-weighted images of 88 PME children and 90 unexposed children. There were no regions or tracts showing significant differences when accounting for multiple comparisons.

### Structural pairwise connectivity

Differences in structural connectivity were measured in terms of fibre count in 88 PME children and 90 unexposed children; 73 connections were significantly different in PME versus unexposed children including 51 with greater connectivity and 22 with lower connectivity in PME children. However, these did not maintain significance after correcting for multiple comparisons. The data are visualized in Supplementary Fig. 3.

### Structural graph network measures

Differences in structural graph network measures were measured in 88 PME children and 90 unexposed children. None of the global graph network metrics showed significant differences. There were regional differences in graph network measures in local efficiency for three regions, betweenness centrality for six regions and clustering coefficient for six regions. However, the significance for these metrics was not sustained when corrected for multiple comparisons. The data are summarized in Supplementary Table 2.

### Connectivity in independent component analysis functional networks

Brain rs-fMRI images were available for 152 subjects—76 PME and 76 unexposed children. Voxelwise clusters were compared between PME and unexposed controls. Clusters under five voxels in size were discarded. There were no regions showing significant differences between the PME and control groups.

### Functional pairwise connectivity

Brain rs-fMRI images were obtained for 152 subjects—76 PME and 76 unexposed children. Brain functional connectivity was assessed between 106 ROIs. Whilst 511 connections showed significance for individual comparisons, significance was not maintained after correcting for multiple comparisons for all connections. This is shown in Supplementary Fig. 4.

### Functional graph network measures

Differences in structural graph network measures were measured in 76 PME children and 76 unexposed children. None of the global graph network metrics showed significant differences. There were regional differences in graph network measures in local efficiency for two regions, betweenness centrality for three regions and clustering coefficient for three regions. However, the significance for these metrics was not sustained when corrected for multiple comparisons. The data are summarized in Supplementary Table 3.

### Prediction of behavioural measures based on graph networks and PME

**Figure 1:**
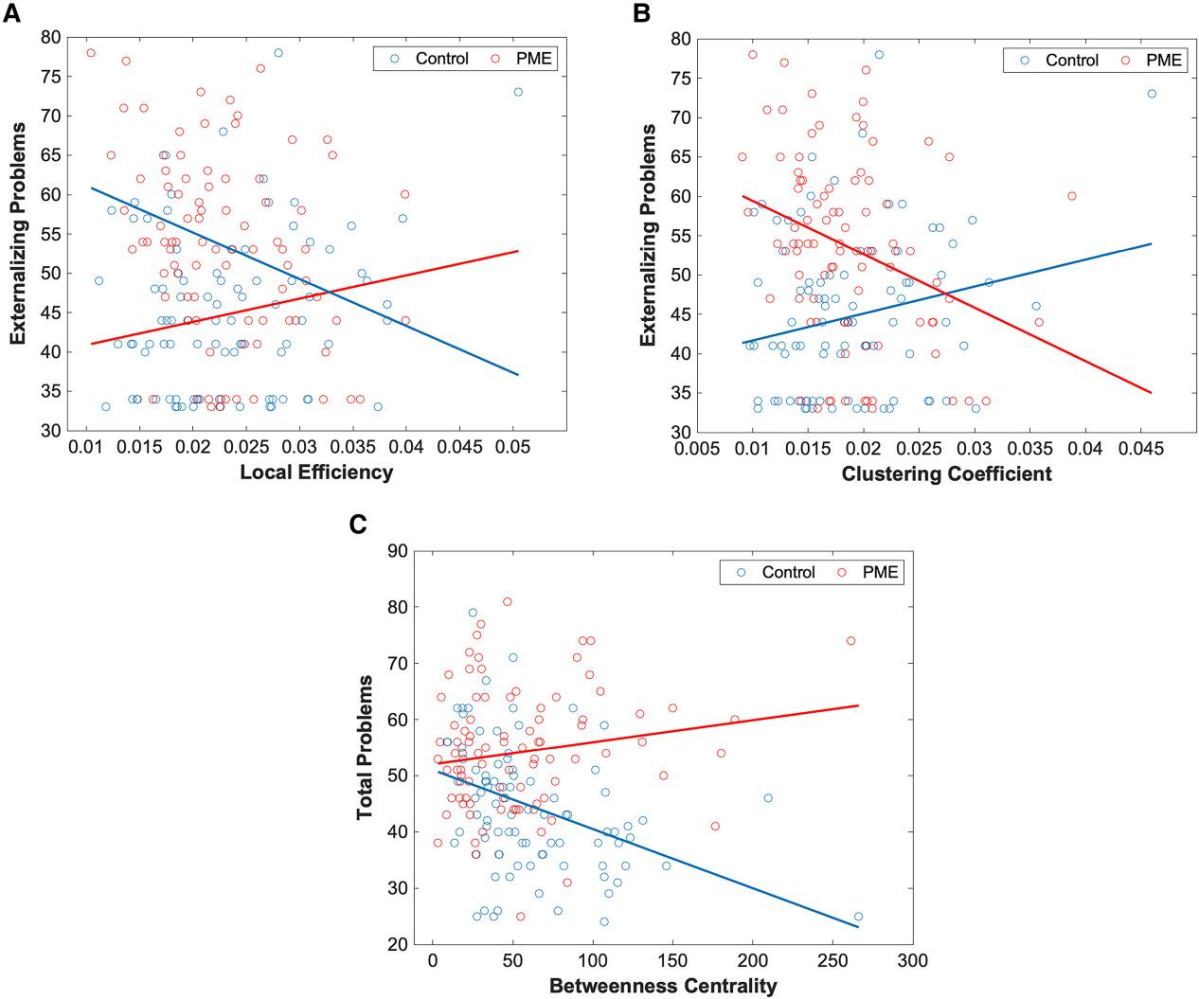
**PME interactions for structural graph networks.** Plots of PME–graph network interactions for structural connectivity using a linear model (*n* = 178). (**A**) Chart represents a scatter plot with raw data overlayed with an interaction plot based on adjusted data for externalizing problems scores versus local efficiency in the right lateral OFC grouped by exposure. There were significant differences in interactions between PME and control groups (*t* = −3.69). (**B**) Chart represents a scatter plot with raw data overlayed with an interaction plot based on adjusted data for externalizing problems scores versus clustering coefficient in the right lateral OFC grouped by exposure. There were significant differences in interactions between PME and control groups (*t* = −3.68). (**C**) Chart represents a scatter plot with raw data overlayed with an interaction plot based on adjusted data for total problems scores versus betweenness centrality in the right amygdala grouped by exposure. There were significant differences in interactions between PME and control groups (*t* = 3.89). A false discovery rate less than 0.05 was considered significant.

We assessed the predictive value of graph network measures on behavioural scores using linear models. For this, we assess the significance of network–PME interactions for nine behavioural scores that demonstrated significant differences between PME and unexposed controls. In our predictive interaction plot, differences in slopes between PME and controls represent different effects of graph network measures on behavioural scores. For structural networks, there was a significant difference in PME–network interactions in local efficiency (Fig. 1A) and clustering coefficient in the right lateral orbitofrontal cortex (OFC) (Fig. 1B) for the externalizing problems scale. Additionally, there was a significant difference in PME–network interactions in betweenness centrality in the right amygdala for the total problems scale (Fig. 1C). Full data can be found in Supplementary Tables 4–12.

**Figure 2:**
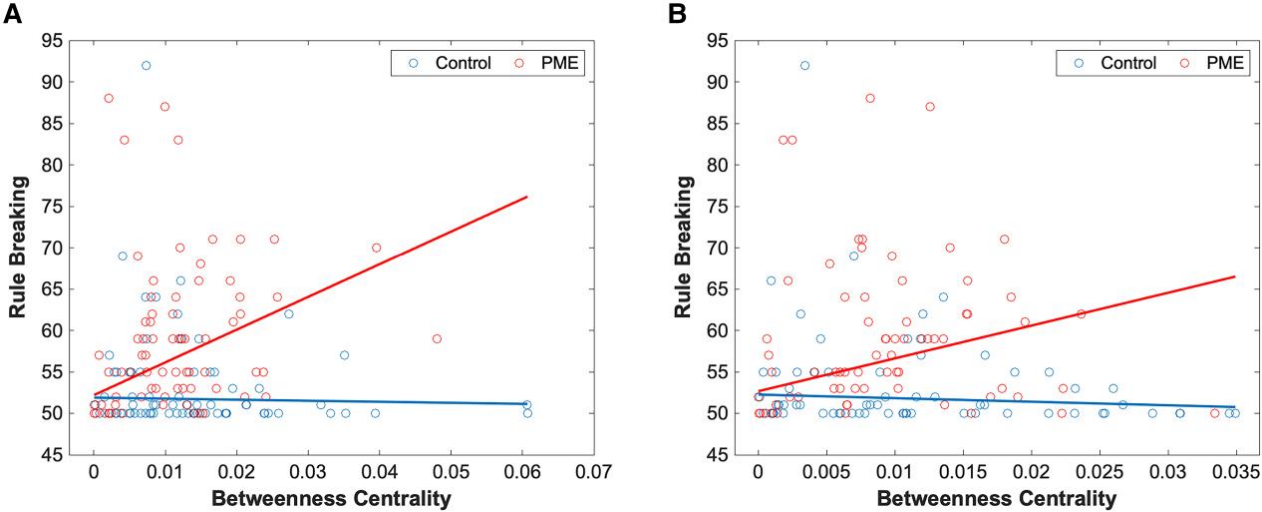
**PME interactions for rule-breaking scores.** Plots of PME–graph network interactions for functional connectivity using a robust linear model (*n* = 152). (**A**) Chart represents a scatter plot with raw data overlayed with an interaction plot based on adjusted data for rule-breaking scores versus betweenness centrality in the right fusiform gyrus grouped by exposure. There were significant differences in interactions between PME and control groups (*t* = 5.02). (**B**) Chart represents a scatter plot with raw data overlayed with an interaction plot based on adjusted data for rule-breaking scores versus betweenness centrality in the left cuneus grouped by exposure. There were significant differences in interactions between PME and control groups (*t* = 5.89). A false discovery rate less than 0.05 was considered significant.

**Figure 3:**
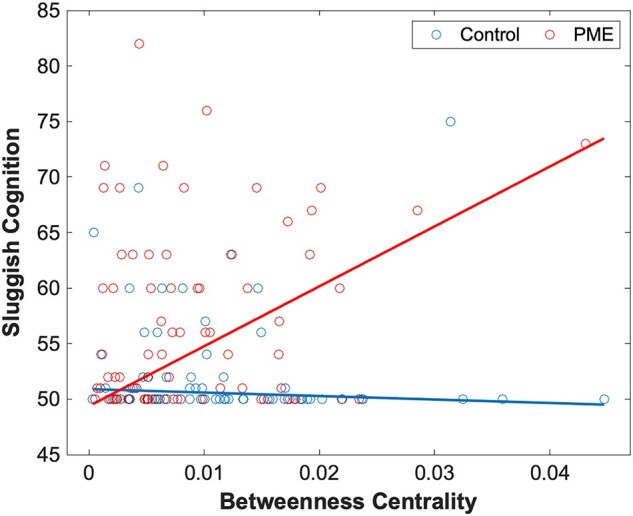
**Sluggish cognition and betweenness centrality.** Plot of PME–graph network interactions for functional connectivity using a robust linear model (*n* = 152). Chart represents a scatter plot with raw data overlayed with an interaction plot based on adjusted data for sluggish cognition scores versus betweenness centrality in the left calcarine sulcus grouped by exposure. There were significant differences in interactions between PME and control groups (*t* = 6.68). A false discovery rate less than 0.05 was considered significant.

**Figure 4:**
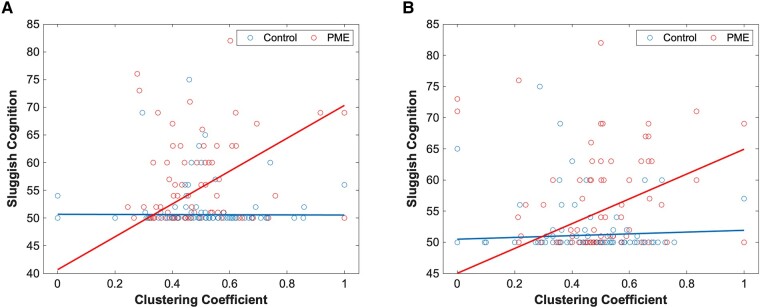
**Sluggish cognition and clustering coefficient.** Plots of PME–graph network interactions for functional connectivity using a robust linear model (*n* = 152). (**A**) Chart represents a scatter plot with raw data overlayed with an interaction plot based on adjusted data for sluggish cognition scores versus clustering coefficient in the left anterior middle temporal gyrus grouped by exposure. There were significant differences in interactions between PME and control groups (*t* = 6.29). (**B**) Chart represents a scatter plot with raw data overlayed with an interaction plot based on adjusted data for sluggish cognition scores versus clustering coefficient in the left anterior inferior temporal gyrus grouped by exposure. There were significant differences in interactions between PME and control groups (*t* = 3.69). A false discovery rate less than 0.05 was considered significant.

**Figure 5:**
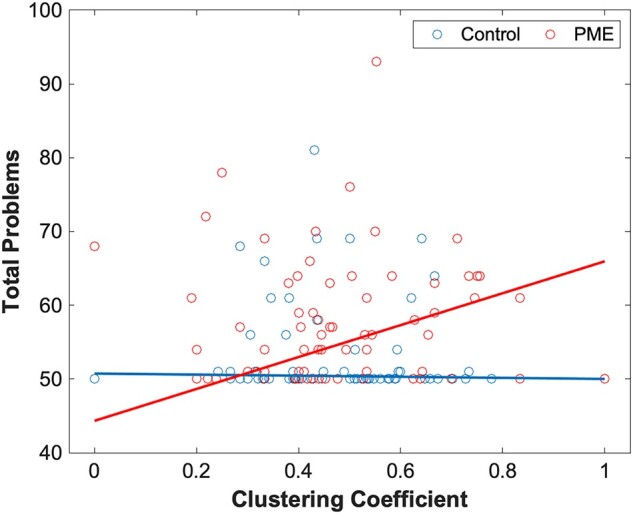
**PME interactions for total problems scores.** Plot of PME–graph network interactions for functional connectivity using a robust linear model (*n* = 152). Chart represents a scatter plot with raw data overlayed with an interaction plot based on adjusted data for total problems scores versus clustering coefficient in the posterior cingulate cortex grouped by exposure. There were significant differences in interactions between PME and control groups (*t* = 4.71). A false discovery rate less than 0.05 was considered significant.

A similar assessment was performed for functional graph networks. Here, there was a significant difference in PME–network interactions in betweenness centrality in the right inferior temporal gyrus (Fig. 2A) and the left cuneus (Fig. 2B) for the rule-breaking behaviour scale. There were also several significant PME–network interactions that were significant predictors for sluggish cognition. Networks where this was observed include betweenness centrality in the left calcarine sulcus (Fig. 3), clustering coefficient in the left middle gyrus (Fig. 4A) and clustering coefficient in the left inferior temporal gyrus (Fig. 4B). Finally, PME–network interactions for the clustering coefficient in the posterior cingulate cortex were a significant predictor for total problems (Fig. 5). Full data can be found in Supplementary Tables 13–21.

## Discussion

This study assesses the impact of PME on behaviour and neurodevelopment in subjects from the Adolescent Brain Cognitive Development database. Overall, PME was a significant contributor to worse behavioural scores in 16 out of 17 CBCL scales, with the somatic complaint scale being the only exception. In this analysis, none of the other factors (age, sex, maternal education and partner education) were significant predictors for behavioural outcomes. Based on clinical thresholds, 9 out of 17 behavioural scales were significantly influenced by PME, after correcting for multiple comparisons. Effected scales were related to attention and behavioural problems. PME was less strongly associated with internalizing factors such as anxiety and depression. Our results are concordant with prior findings showing PME being associated with a higher risk of developing behavioural problems and ADHD.^11,77^

Despite significant differences in behavioural scores between adolescents with PME and non-PME controls, differences in neuroimaging measurements were not statistically significant. There were no significant differences in brain structure on both voxel-based and surface-based morphometry after multiple comparison corrections. Similarly, there were no significant differences in FA, mean diffusivity, axial diffusivity or radial diffusivity. However, other studies have shown that children with PME demonstrated greater thickness in the frontal cortices^78^ and alterations in brain volume. These different results may be related to different ages at evaluation, socioenvironmental confounders or image analysis methods.^79^

Results for differences in structural and functional connectivity were mixed. We assessed connectivity using both ROI-to-ROI and graph network connectivity. ROI-to-ROI connectivity involves direct connections, either anatomical or functional, from one region to another. On the other hand, graph networks are organizational frameworks involving nodes and edges that are centred around a particular brain region or the whole brain. From our results, there were no significant differences of ROI-to-ROI or graph network connectivity based on the presence of PME. However, several PME–graph network interactions were significant predictors for multiple behavioural scales.

Graph network metrics that were involved with significant differences between PME and control groups include clustering coefficient, local efficiency and betweenness centrality. All significant metrics were significant at the nodal level (anatomical ROI). Clustering coefficient is a measure of how nodes tend to cluster together. At the level of a node, or brain region, this is the proportion of connections that are being utilized by its neighbouring regions compared with the total number of possible connections.^80^ Local efficiency is involved with how well information is exchanged between its neighbouring regions in the absence of the node.^81^ Increased levels of both indicate greater connectivity. Betweenness centrality is a metric that indicates the amount of influence a region has for the overall network. This is assessed by the number of shortest paths that travel through the brain region.^82^

There were several structural graph networks that had significantly different correlations with behavioural scores based on the presence of PME. Correlations between graph network metrics (clustering coefficient and local efficiency) of the right lateral OFC with externalizing problems in the PME versus control groups. This indicates that local connectivity of the right lateral OFC and neighbouring regions may have different association to externalizing problems in those with PME. The OFC is an important structure for decision-making and the lateral OFC has been linked to conforming to social values,^83^ suppression of responses,^84^ evaluation of punishment^85^ and devaluation.^86^ Another node that showed differences in correlation of structural metrics with behavioural scores was betweenness centrality of the right amygdala for the total problems scale. This suggests that the influence of the right amygdala may have a different association with total problems scores in those with PME compared with controls. The amygdala has been associated with emotions such as fear^87-89^ and memory^89,90^ and is a component of the rewards network.^91,92^ The amygdala is also functionally connected to the OFC,^93^ and this connection is shown to be responsible for with social anxiety,^94,95^ immediate rewards,^96^ goal-directed behaviour^84,97^ and reinforcement.^98^ Additionally, betweenness centrality in the amygdala has been associated with increased stress.^34^

Our results suggest that PME differentially impacts the association of several functional graph networks and behavioural scores. First, we identified differences in correlations of betweenness centrality in the right fusiform gyrus and left cuneus with the rule-breaking scale in those with PME compared with controls. The fusiform gyrus is an important region for facial recognition, and connectivity in this region has been linked to learning disabilities such as dyslexia.^99^ The cuneus is involved with visual processing and atypical connectivity in this region has been linked with autism spectrum disorders.^100^ Increased significance of these regions in those specifically with PME is noteworthy.

Second, we identified differences in associations of functional graph network metrics (betweenness centrality in the left calcarine sulcus and clustering coefficient in the left anterior middle and inferior temporal gyri) with the sluggish cognition scale. Increased influence of the left calcarine sulcus is associated with poorer outcomes of sluggish cognition in those with PME compared with controls. Reduced connectivity in the neighbouring regions may imply that these systems are more poorly regulated. The calcarine sulcus is a prominent component in the visual cortex and functional connectivity in the region has been linked with eye misalignment.^101^ The calcarine sulcus may play a more significant role in cognition in those with PME.

Increased regional connectivities in the left anterior middle and inferior temporal gyri were associated with poorer outcomes of sluggish cognition in those with PME compared with controls. The middle and inferior temporal gyri are involved with semantic processing and visual perception. The association between increased regional connectivity and increased symptoms, even if the overall correlation is weak, is somewhat unexpected. Greater connectivity would typically be associated with improved functioning. However, it is possible that this greater regional connectivity could be a form of overcompensation for functional deficits.

Finally, we identified differences associations of the functional graph network clustering coefficient in the posterior cingulate cortex with the total problems scale in PME versus controls. The posterior cingulate cortex is a highly connected region that is an important region of the default mode network. Considering that the default mode network is associated with mind wandering,^102^ increased clustering coefficient may be associated with sluggish outcomes. However, this effect was notable in the PME population and not the controls. Altered connectivity in the default mode network has also been linked to many disorders including ADHD,^103^ depression,^104^ schizophrenia^105^ and other mental disorders.

In those with PME, there was a significant association of multiple graph measures in regions involving visual processing with behavioural outcomes. This is unsurprising given that PME has been linked with impaired visual processing in infants and toddlers.^106^ This may contribute to impaired learning as children with PME can increase the risk of learning disabilities and ADHD.^9-12,107^

Other studies have demonstrated important findings on the short- and long-term impact of PME. The Ottawa Prenatal Prospective Study found that PME impacted neurocognition at various stages of life.^13^ Whilst no correlation between PME and overall IQ was found, the study did find that children with PME tend to score more poorly on tasks related to executive functioning. Neonates with PME demonstrated decreased visual habituation and increased tremors. As children, the PME group had poorer verbal skills, memory, attention, visual perception and executive functioning. Similar issues seemed to continue into adulthood as they exhibited reduced concentration and inhibition along with impaired visual memory analytical skills.^9,13^ From this study, 31 subjects underwent fMRI using four tasks^15^ with the results demonstrating that the PME group had increased brain activity in left posterior brain regions for similar tasks compared with unexposed controls.

Other studies have shown differences in structural and functional connectivity in infants with PME.^21,108^ The lack of persistence of these structural and functional alterations may be at least partly explained by developmental neuroplasticity and postnatal factors. However, we identified differences in associations of multiple structural and functional graph network measures for behavioural scores based on the presence of PME. This would suggest that despite neuroplasticity, there may be some persistent focal developmental impacts of prenatal marijuana on the brain.

There have been other studies examining the long-term effects of PME based on subjects with the ABCD database. Based on a larger sample size from the ABCD® Study, differences in behavioural outcomes were observed in subjects with PME.^109^ Additionally, in the same study, significantly lower brain volumes were reported after multiple comparison corrections. Positive confirmatory findings for brain volume, which were not found in our study, may be due to a larger sample size and greater statistical power. Another study by Cioffredi *et al*.^110^ looked at PME in the context of three fMRI tasks and CBCL scores from the ABCD® Study. Similar to our study, they identified adolescents with PME having greater attention, externalizing and total problems scores. Additionally, they did not observe differences in terms of cognitive performance or patterns of brain activation during tasks. This falls in line with our results in that there were significant differences in behavioural scores, particularly externalizing scores, but there were no confirmatory findings regarding differences in MRI measurements.

Overall, this study showed a significant influence of PME on behavioural scores for 16 out of 17 scales and clinical categories for 9 out of 17 scales. Furthermore, several PME–graph network interactions were identified as significant predictors for multiple behavioural scales. This indicates that network measures in these regions could be biomarkers for those with PME who may be at risk for developmental and behavioural problems. On the other hand, we were not able to find a significant difference in anatomical measurements or brain connectivity between the PME and control groups after correcting for multiple comparisons. However, other studies have shown significant differences in these factors. Additionally, considering that there were several interactions terms that were significant for multiple behavioural scales, it is likely that PME has some effect on connectivity even though these could not pass the statistical threshold in our study due to limitations due to retrospective nature and probable undetermined environmental effects in the ABCD data.

There are several limitations that need to be noted. For the ABCD database, the proportion of adolescents with PME was relatively small, and being a retrospective assessment, there may be recall bias. For future studies, we would recommend using a prospective design and follow-up of a larger cohort tracking children from the neonatal stage to adolescence to better assess the causality of PME on developmental conditions such as behavioural problems or ADHD and more accurately assess brain developmental alterations. Whilst we assessed the differences between PME and unexposed children, we did not account for the amount of cannabis used. Although we had 178 subjects with behavioural scores, only 152 subjects had fMRI data. We used different atlases for different imaging modalities. For structural connectivity, we used the automated anatomical labelling version 2 atlas, and for functional connectivity, we used the Harvard–Oxford atlas. Since we did not correlate data from the different imaging modalities, there is limited effect for these differences. Finally, this study also did not take into account postnatal effects, as these have also shown to impact behavioural outcomes for children with prenatal substance exposure.^111^

## Conclusions

This study, based on images and data from the Adolescent Brain Cognitive Development study, assessed the impact of PME on neurodevelopment in pre-adolescent children. There were significant differences in multiple CBCL scales in PME children compared with unexposed controls. There were no statistically significant differences in anatomical measurements and brain connectivity between the PME and control groups. However, there were PME–graph network interactions that were significant for behavioural scores. This suggests that altered brain networks may underlie behavioural outcomes in adolescents with PME. More work needs to be conducted to better understand the prognostic value of brain structural and functional network measures in PME.

## Supplementary Material

fcae001_Supplementary_Data

## Data Availability

Data for this study were obtained from ABCD® Study (https://abcdstudy.org), held in the NIMH Data Archive.^35,36^
