# Supplementary material for: Impact of prenatal marijuana exposure on adolescent brain structural and functional connectivity and behavioural outcomes
Source: Brain Commun. 2024 Jan 8;6(2):fcae001. doi: 10.1093/braincomms/fcae001 (PMC10914455; doi:10.1093/braincomms/fcae001)
Supplement: fcae001_Supplementary_Data [file fcae001_supplementary_data.pdf]

Supplementary Material

	PME	Control	p-value	p-FDR
Anxiety/Depression			0.316	0.335
Borderline	6	4		
Clinical	8	4		
No Issue	74	82		
Withdrawal/Depression			0.185	0.225
Borderline	7	3		
Clinical	4	1		
No Issue	77	86		
Somatic Complaints			0.230	0.261
Borderline	7	6		
Clinical	6	4		
No Issue	75	80		
Social Problems			0.109	0.16
Borderline	5	2		
Clinical	5	1		
No Issue	78	87		
Thought Problems			0.002	0.02*
Borderline	8	0		
Clinical	8	4		
No Issue	72	86		
Attention Problems			0.006	0.025*
Borderline	6	2		
Clinical	9	1		
No Issue	73	87		
Rule-breaking Behavior			0.021	0.042*
Borderline	7	1		
Clinical	6	2		
No Issue	75	87		
Aggressive Behavior			0.021	0.042*
Borderline	11	3		
Clinical	6	2		
No Issue	71	85		
Internalizing Problems			0.113	0.16
Borderline	12	8		
Clinical	7	2		
No Issue	69	80		
Externalizing Problems			0.007	0.025*
Borderline	9	2		
Clinical	8	2		

No Issue	71	86		
ADHD			0.002	0.02*
Borderline	10	2		
Clinical	7	1		
No Issue	71	87		
Oppositional Disorder			0.034	0.058
Borderline	3	0		
Clinical	11	5		
No Issue	74	85		
Conduct Disorder			0.022	0.042*
Borderline	8	2		
Clinical	7	2		
No Issue	73	86		
Sluggish Cognition			0.014	0.04*
Borderline	5	0		
Clinical	8	3		
No Issue	75	87		
OCD			0.180	0.225
Borderline	5	3		
Clinical	8	3		
No Issue	75	84		
Stress Problems			0.371	0.371
Borderline	6	6		
Clinical	6	2		
No Issue	76	82		
Total Problems			0.007	0.025*
Borderline	6	1		
Clinical	9	2		
No Issue	73	87		

*Denotes significance

Supplementary Table 1: Child behavioral checklist clinical classifications based on sub-scale scores for prenatal marijuana exposure (PME) and unexposed children. Comparisons were calculated using a Fisher's exact test and multiple comparison correction was based on false discovery rate. A false discovery rate less than 0.05 was considered significant.

	t-stat	p-value	p-FDR
Local Efficiency			
Olfactory - L	2.01	0.046	0.738
Posterior OFC - R	-2.38	0.019	0.738
Inferior parietal - L	2.00	0.047	0.738
Betweenness Centrality			
Inferior frontal operculum - R	2.17	0.031	0.738
Cuneus - R	2.49	0.014	0.738
Inferior occipital - L	2.26	0.025	0.738
Precuneus - L	2.06	0.041	0.738
Thalamus - L	-2.13	0.035	0.738
Temporal pole - L	-2.21	0.029	0.738
Clustering Coefficient			
Inferior frontal operculum - R	-2.03	0.044	0.738
Posterior OFC - R	-2.41	0.017	0.738
Calcarine – R	-2.23	0.027	0.738
Lingual_R	-2.00	0.047	0.738
Precuneus - L	-2.13	0.034	0.738
Thalamus - L	2.08	0.039	0.738

Supplementary Table 2: A linear regression was performed with age, sex, PME, maternal college, and paternal college as predictor variables and structural network measures as the response variables. Data in table shows predictive value of PME on network measures for p-value < 0.05. A false discovery rate less than 0.05 was considered significant. None of the values maintained significance after correcting for multiple comparisons.

	t-stat	p-value	p-FDR
Local Efficiency			
Sup lateral occipital - R	-2.16	0.033	0.987
Thalamus - L	-2.06	0.042	0.987
Betweenness Centrality			
Ant inferior temporal gyrus - L	-2.02	0.045	0.987
Supp motor area	-1.99	0.049	0.987
Supracalcarine cortex - L	-2.16	0.033	0.987
Clustering Coefficient			
Insula - R	-2.23	0.027	0.987
Ant superior temporal gyrus - R	-2.81	0.006	0.987
Orbitofrontal cortex - R	-2.19	0.030	0.987

Supplementary Table 3: A linear regression was performed with age, sex, PME, maternal college, and paternal college as predictor variables and functional network measures as the response variables. Data in table shows predictive value of PME on network measures for p-value < 0.05. A false discovery rate less than 0.05 was considered significant. None of the values maintained significance after correcting for multiple comparisons.

	t-stat	p-value	p-FDR
Local Efficiency			
Superior temporal - R	2.08	0.039	0.826
Sup temporal pole - L	2.00	0.047	0.826
Betweenness Centrality			
Amygdala - R	3.03	0.003	0.793
Thalamus - R	-2.02	0.045	0.826
Superior temporal - R	-2.31	0.022	0.826
Clustering Coefficient			
Medial OFC - L	2.06	0.041	0.826
Anterior OFC - L	2.08	0.039	0.826
Posterior OFC - L	2.03	0.044	0.826
Sup orbital - L	2.08	0.039	0.826
Sup occipital - R	2.18	0.031	0.826
Inferior occipital - L	-1.99	0.048	0.826
Superior temporal - R	2.31	0.022	0.826
Sup temporal pole - L	2.57	0.011	0.826

Supplementary Table 4: Structural graph network measures correlated with CBCL thought problems scale based on a general linear model. Data showing PME-network interaction terms with p-values < 0.05. A false discovery rate less than 0.05 was considered significant. None of the values maintained significance after correcting for multiple comparisons.

	t-stat	p-value	pFDR
Local Efficiency			
Olfactory - L	2.18	0.031	0.724
Posterior cingulate - R	2.67	0.008	0.724
Cuneus - L	2.23	0.027	0.724
Sup orbital - L	2.38	0.018	0.724
Heschl - R	1.99	0.049	0.724
Sup temporal pole - L	2.72	0.007	0.724
Temporal pole - L	2.21	0.028	0.724
Betweenness Centrality			
Pallidum - L	2.05	0.042	0.724
Clustering Coefficient			
Olfactory - L	2.13	0.035	0.724
Posterior cingulate - R	2.41	0.017	0.724
Calcarine - R	2.33	0.021	0.724
Cuneus - L	2.07	0.040	0.724
Sup orbital - L	2.48	0.014	0.724
Sup temporal pole - L	2.90	0.004	0.724

Supplementary Table 5: Structural graph network measures correlated with CBCL attention problems scale based on a general linear model. Data showing PME-network interaction terms with p-values < 0.05. A false discovery rate less than 0.05 was considered significant. None of the values maintained significance after correcting for multiple comparisons.

	t-stat	p-value	pFDR
Local Efficiency			
Lateral OFC - R	-3.56	0.000	0.068
Posterior cingulate - R	2.41	0.017	0.830
Hippocampus - R	2.08	0.039	0.830
Heschl - R	2.38	0.018	0.830
Temporal pole - L	2.07	0.040	0.830
Temporal Pole - R	2.13	0.035	0.830
Betweenness Centrality			
Amygdala - R	2.55	0.012	0.830
Caudate - L	2.81	0.005	0.517
Inferior temporal - R	-1.98	0.049	0.830
Clustering Coefficient			
Lateral OFC - R	-3.68	0.000	0.068
Posterior cingulate - R	2.08	0.039	0.830

Supplementary Table 6: Structural graph network measures correlated with CBCL rule-breaking behavior scale based on a general linear model. Data showing PME-network interaction terms with p-values < 0.05. A false discovery rate less than 0.05 was considered significant. None of the values maintained significance after correcting for multiple comparisons.

	t-stat	p-value	pFDR
Local Efficiency			
Supp motor area - R	1.98	0.049	0.973
Lateral OFC - R	-3.32	0.001	0.218
Heschl - R	2.08	0.039	0.973
Betweenness Centrality			
Rectus - L	-2.07	0.040	0.973
Amygdala - R	3.08	0.002	0.227
Clustering Coefficient			
Supp motor area - R	2.16	0.032	0.973
Lateral OFC - R	-3.22	0.002	0.218
Superior temporal - R	2.26	0.025	0.973

Supplementary Table 7: Structural graph network measures correlated with CBCL aggressive behavior scale based on a general linear model. Data showing PME-network interaction terms with p-values < 0.05. A false discovery rate less than 0.05 was considered significant. None of the values maintained significance after correcting for multiple comparisons.

	t-stat	p-value	pFDR
Global Efficiency	2.00	0.047	0.808
Local Efficiency			
Supp motor area - R	2.25	0.026	0.808
Lateral OFC - R	-3.69	0.000	0.044*
Hippocampus - R	2.23	0.027	0.808
Supramarginal gyrus - R	2.00	0.047	0.808
Heschl - R	2.00	0.047	0.808
Superior temporal - R	2.25	0.025	0.808
Temporal pole - L	2.32	0.021	0.808
Betweenness Centrality			
Amygdala - R	3.27	0.001	0.123
Clustering Coefficient			
Supp motor area - R	2.20	0.029	0.808
Lateral OFC - R	-3.68	0.000	0.044*
Hippocampus - R	1.98	0.049	0.808
Superior temporal - R	2.25	0.026	0.808

* Denotes significance

Supplementary Table 8: Structural graph network measures correlated with CBCL externalizing problems scale based on a general linear model. Data showing PME-network interaction terms with p-values < 0.05. A false discovery rate less than 0.05 was considered significant. Two measures, local efficiency and betweenness centrality in right lateral orbitofrontal cortex, show significance after correcting for multiple comparisons.

	t-stat	p-value	pFDR
Local Efficiency			
Medial OFC - L	1.98	0.050	0.876
Lateral OFC - R	-2.72	0.007	0.876
Posterior cingulate - R	2.52	0.013	0.876
Sup orbital - L	2.26	0.025	0.876
Heschl - R	1.99	0.048	0.876
Sup temporal pole - L	2.01	0.046	0.876
Temporal pole - L	2.44	0.016	0.876
Betweenness Centrality			
Med orbital SFG - R	2.06	0.041	0.876
Clustering Coefficient			
Medial OFC - L	2.41	0.017	0.876
Lateral OFC - R	-2.35	0.020	0.876
Posterior cingulate - R	2.16	0.032	0.876
Sup orbital - L	2.39	0.018	0.876
Sup temporal pole - L	2.02	0.045	0.876
Temporal pole - L	2.24	0.026	0.876

Supplementary Table 9: Structural graph network measures correlated with CBCL ADHD scale based on a general linear model. Data showing PME-network interaction terms with p-values < 0.05. A false discovery rate less than 0.05 was considered significant. None of the values maintained significance after correcting for multiple comparisons.

	t-stat	p-value	pFDR
Local Efficiency			
Supp motor area - R	2.14	0.034	0.951
Lateral OFC - R	-3.41	0.001	0.113
Posterior cingulate - L	2.08	0.039	0.951
Posterior cingulate - R	2.27	0.025	0.951
Heschl - R	2.31	0.022	0.951
Temporal pole - L	2.15	0.033	0.951
Betweenness Centrality			
Rectus - L	-1.98	0.049	0.951
Amygdala - R	3.16	0.002	0.175
Caudate - L	2.12	0.035	0.951
Inferior temporal - R	-2.09	0.038	0.951
Clustering Coefficient			
Supp motor area - R	2.09	0.038	0.951
Lateral OFC - R	-3.56	0.000	0.113

Supplementary Table 10: Structural graph network measures correlated with CBCL conduct disorder scale based on a general linear model. Data showing PME-network interaction terms with p-values < 0.05. A false discovery rate less than 0.05 was considered significant. None of the values maintained significance after correcting for multiple comparisons.

	t-stat	p-value	pFDR
Local Efficiency			
Insula - L	2.16	0.032	0.775
Posterior cingulate - R	2.00	0.047	0.775
Heschl - R	2.24	0.027	0.775
Sup temporal pole - L	3.26	0.001	0.189
Sup temporal pole - R	2.11	0.037	0.775
Betweenness Centrality			
Putamen - L	2.05	0.042	0.775
Clustering Coefficient			
Insula - L	2.35	0.020	0.775
Posterior cingulate - R	2.03	0.044	0.775
Sup temporal pole - L	3.52	0.001	0.159
Sup temporal pole - R	2.18	0.031	0.775

Supplementary Table 11: Structural graph network measures correlated with CBCL sluggish cognition scale based on a general linear model. Data showing PME-network interaction terms with p-values < 0.05. A false discovery rate less than 0.05 was considered significant. None of the values maintained significance after correcting for multiple comparisons.

	t-stat	p-value	pFDR
Local Efficiency			
Precentral gyrus - L	2.32	0.022	0.676
Sup orbital - L	2.26	0.025	0.676
Caudate - L	2.22	0.028	0.676
Heschl - R	2.17	0.032	0.676
Superior temporal - R	2.54	0.012	0.676
Sup temporal pole - L	2.28	0.024	0.676
Temporal pole - L	2.37	0.019	0.676
Betweenness Centrality			
Amygdala - R	3.89	0.000	0.041*
Pallidum - L	2.28	0.024	0.676
Superior temporal - R	-2.15	0.033	0.676
Clustering Coefficient			
Posterior OFC - R	2.25	0.026	0.676
Superior temporal - R	2.62	0.010	0.676
Sup temporal pole - L	2.46	0.015	0.676
Sup temporal pole - R	2.20	0.029	0.676

* Denotes significance

Supplementary Table 12: Structural graph network measures correlated with CBCL total problems scale based on a general linear model. Data showing PME-network interaction terms with p-values < 0.05. A false discovery rate less than 0.05 was considered significant. Betweenness centrality in the right amygdala showed significant differences after correcting for multiple comparisons.

	t-stat	p-value	pFDR
Local Efficiency			
Accumbens - R	-2.42	0.017	0.995
Betweenness Centrality			
Precuneus	2.26	0.025	0.995

Supplementary Table 13: Functional graph network measures correlated with CBCL thought problems scale based on a robust linear regression. Data showing PME-network interaction terms with p-values < 0.05. A false discovery rate less than 0.05 was considered significant. None of the values maintained significance after correcting for multiple comparisons.

	t-stat	p-value	pFDR
Local Efficiency			
Frontal pole - R	-2.51	0.013	0.404
Pars opercularis - L	-2.33	0.021	0.487
Precentral gyrus - R	-2.02	0.046	0.680
Precentral gyrus - L	-1.99	0.049	0.680
Middle temporal gyrus - L	2.53	0.012	0.404
Inferior temporal gyrus - L	2.07	0.040	0.638
Angular gyrus - R	-2.17	0.032	0.533
Orbitofrontal cortex - R	-2.70	0.008	0.404
Orbitofrontal cortex - L	-2.89	0.004	0.404
Frontal operculum - R	-2.83	0.005	0.404
Parietal operculum - L	-2.59	0.010	0.404
Occipital pole - L	1.99	0.050	0.680
Betweenness Centrality			
Frontal pole - R	2.53	0.012	0.404
Insula - R	-2.35	0.020	0.487
Fusiform gyrus - R	2.27	0.025	0.529
Supramarginal gyrus - L	2.49	0.014	0.404
Calcarine sulcus - R	2.19	0.030	0.533
Precuneus	2.54	0.012	0.404
Supracalcarine cortex - R	2.45	0.015	0.412
Thalamus - R	2.60	0.010	0.404
Clustering Coefficient			
Frontal pole - R	-2.24	0.027	0.531
Frontal operculum - R	-2.68	0.008	0.404
Occipital pole - L	2.23	0.029	0.533

Supplementary Table 14: Functional graph network measures correlated with CBCL attention problems scale based on a robust linear regression. Data showing PME-network interaction terms with p-values < 0.05. A false discovery rate less than 0.05 was considered significant. None of the values maintained significance after correcting for multiple comparisons.

	t-stat	p-value	pFDR
Local Efficiency			
Pars triangularis - R	-2.37	0.019	0.301
Precentral gyrus - R	-2.64	0.009	0.279
Precentral gyrus - L	-2.04	0.043	0.419
Temporal pole - L	-1.98	0.050	0.420
Post superior temporal gyrus - R	2.09	0.038	0.419
Post inferior temporal gyrus - R	-2.36	0.020	0.301
Supramarginal gyrus - L	2.24	0.026	0.368
Supp motor area	-2.68	0.008	0.279
Subcallosal cortex	-2.68	0.008	0.279
Cuneus	2.03	0.045	0.419
Orbitofrontal cortex - R	-1.98	0.049	0.420
Frontal operculum - R	-2.44	0.016	0.279
Parietal operculum - L	-2.45	0.016	0.279
Planum temporale - R	-3.03	0.003	0.178
Occipital pole - R	2.18	0.032	0.406
Pallidum - L	-2.95	0.004	0.178
Accumbens - R	-2.05	0.042	0.419
Accumbens - L	-2.37	0.019	0.301
Betweenness Centrality			
Frontal pole - R	2.03	0.044	0.419
Precentral gyrus - R	2.17	0.031	0.406
Ant superior temporal gyrus - L	2.08	0.039	0.419
Fusiform gyrus - R	5.02	0.000	<0.001*
Calcarine sulcus - R	2.45	0.016	0.279
Calcarine sulcus - L	2.48	0.014	0.279
Medial prefrontal cortex	1.99	0.048	0.420
Precuneus	3.01	0.003	0.178
Cuneus - L	5.89	0.000	<0.001*
Pallidum - L	2.01	0.046	0.419
Clustering Coefficient			
Pars triangularis - R	-2.46	0.015	0.279
Precentral gyrus - R	-3.21	0.002	0.175
Ant inferior temporal gyrus - L	2.54	0.012	0.279
Medial prefrontal cortex	-2.27	0.025	0.363
Supp motor area	-2.09	0.039	0.419
Orbitofrontal cortex - R	-2.02	0.045	0.419
Frontal operculum - R	-2.52	0.013	0.279
Occipital pole - R	2.08	0.041	0.419
Pallidum - L	-2.93	0.004	0.178
Accumbens - L	-2.55	0.012	0.279

* Denotes significance

Supplementary Table 15: Functional graph network measures correlated with CBCL rule-breaking behavior scale based on a robust linear regression. Data showing PME-network interaction terms with p-values < 0.05. A false discovery rate less than 0.05 was considered significant. Betweenness centrality in the right inferior temporal gyrus and left cuneus showed significant differences after correcting for multiple comparisons.

	t-stat	p-value	pFDR
Local Efficiency			
Insula - R	-2.45	0.015	0.990
Sup parietal lobule - L	-2.44	0.016	0.990
Lingual gyrus - L	-2.17	0.032	0.990
Betweenness Centrality			
Post middle temporal gyrus - R	2.82	0.005	0.990
Postcentral gyrus - R	2.60	0.010	0.990
Sup lateral occipital cortex - L	-2.20	0.030	0.990
Clustering Coefficient			
Sup parietal lobule - L	-2.01	0.046	0.990

Supplementary Table 16: Functional graph network measures correlated with CBCL aggressive behavior scale based on a robust linear regression. Data showing PME-network interaction terms with p-values < 0.05. A false discovery rate less than 0.05 was considered significant. None of the values maintained significance after correcting for multiple comparisons.

	t-stat	p-value	pFDR
Local Efficiency			
Hippocampal gyrus - R	2.72	0.007	0.807
Occipital pole - L	2.45	0.016	0.807
Caudate - L	2.55	0.012	0.807
Betweenness Centrality			
Pars triangularis - R	2.40	0.018	0.807
Ant superior temporal gyrus - L	-2.19	0.030	0.981
Precuneus	2.65	0.009	0.807
Clustering Coefficient			
Frontal pole - L	-2.67	0.009	0.807
Medial prefrontal cortex	-2.12	0.036	0.981
Hippocampal gyrus - R	2.48	0.014	0.807
Occipital pole - L	2.04	0.044	0.981

Supplementary Table 17: Functional graph network measures correlated with CBCL externalizing problems scale based on a robust linear regression. Data showing PME-network interaction terms with p-values < 0.05. A false discovery rate less than 0.05 was considered significant. None of the values maintained significance after correcting for multiple comparisons.

	t-stat	p-value	pFDR
Local Efficiency			
Post superior temporal gyrus - R	2.57	0.011	0.686
Supramarginal gyrus - L	2.28	0.024	0.721
Occipital pole - L	2.16	0.034	0.721
Betweenness Centrality			
Frontal pole - R	2.73	0.007	0.686
Pars triangularis - R	2.20	0.029	0.721
Ant inferior temporal gyrus - L	-2.03	0.044	0.887
Fusiform gyrus - R	2.38	0.018	0.721
Calcarine sulcus - L	2.16	0.033	0.721
Precuneus	2.20	0.029	0.721
Parahippocampal gyrus - L	2.34	0.021	0.721
Parietal operculum - R	2.52	0.013	0.686
Amygdala - L	2.63	0.009	0.686
Clustering Coefficient			
Post superior temporal gyrus - R	2.20	0.029	0.721
Ant inferior temporal gyrus - R	2.70	0.008	0.686
Precuneus	-2.54	0.012	0.686
Occipital pole - L	2.45	0.017	0.721

Supplementary Table 18: Functional graph network measures correlated with CBCL ADHD scale based on a robust linear regression. Data showing PME-network interaction terms with p-values < 0.05. A false discovery rate less than 0.05 was considered significant. None of the values maintained significance after correcting for multiple comparisons.

	t-stat	p-value	pFDR
Local Efficiency			
Insula - L	-2.12	0.035	0.513
Superior frontal gyrus - R	-2.08	0.039	0.513
Occ middle temporal gyrus - R	2.46	0.015	0.488
Post inferior temporal gyrus - L	3.31	0.001	0.192
Supramarginal gyrus - L	2.19	0.030	0.513
Paracingulate gyrus - R	-2.69	0.008	0.488
Lingual gyrus - L	-2.18	0.031	0.513
Thalamus - R	-2.09	0.038	0.513
Thalamus - L	-2.25	0.026	0.513
Betweenness Centrality			
Frontal pole - R	2.09	0.038	0.513
Fusiform gyrus - L	-2.22	0.028	0.513
Post inferior temporal gyrus - R	2.05	0.042	0.513
Calcarine sulcus - L	2.51	0.013	0.488
Medial prefrontal cortex	2.48	0.014	0.488
Precuneus	2.61	0.010	0.488
Parahippocampal gyrus - L	2.82	0.005	0.438
Lingual gyrus - L	3.80	0.000	0.068
Lingual gyrus - L	2.02	0.046	0.541
Parietal operculum - R	2.36	0.020	0.513
Clustering Coefficient			
Superior frontal gyrus - R	-2.07	0.040	0.513
Post inferior temporal gyrus - L	3.14	0.002	0.218
Paracingulate gyrus - R	-2.17	0.032	0.513
Parahippocampal gyrus - L	-2.09	0.039	0.513
Lingual gyrus - L	-2.50	0.014	0.488
Frontal operculum - R	-2.24	0.027	0.513
Occipital pole - L	2.17	0.033	0.513

Supplementary Table 19: Functional graph network measures correlated with CBCL conduct disorder scale based on a robust linear regression. Data showing PME-network interaction terms with p-values < 0.05. A false discovery rate less than 0.05 was considered significant. None of the values maintained significance after correcting for multiple comparisons.

	t-stat	p-value	pFDR
Local Efficiency			
Precentral gyrus - R	-2.83	0.005	0.339
Calcarine sulcus - R	-2.62	0.010	0.390
Subcallosal cortex	-2.48	0.014	0.513
Betweenness Centrality			
Calcarine sulcus - L	6.68	0.000	<0.001*
Parahippocampal gyrus - R	2.68	0.008	0.377
Clustering Coefficient			
Ant middle temporal gyrus - L	6.29	0.000	<0.001*
Ant inferior temporal gyrus - R	2.75	0.007	0.364
Ant inferior temporal gyrus - L	3.69	0.000	0.027*

* Denotes significance

Supplementary Table 20: Functional graph network measures correlated with CBCL sluggish cognition scale based on a robust linear regression. Data showing PME-network interaction terms with p-values < 0.05. A false discovery rate less than 0.05 was considered significant. Measures showing significance after multiple comparisons include betweenness centrality in the left calcarine sulcus, and clustering coefficient the left middle temporal gyrus and left inferior temporal gyrus.

	t-stat	p-value	pFDR
Local Efficiency			
Precentral gyrus - R	-3.45	0.001	0.079
Precentral gyrus - L	-2.10	0.038	0.768
Temporal pole - L	-2.02	0.046	0.768
Inferior temporal gyrus - L	2.46	0.015	0.745
Lat occipital - R	-2.06	0.042	0.768
Posterior cingulate	2.05	0.042	0.768
Parietal operculum - L	-2.26	0.026	0.745
Betweenness Centrality			
Frontal pole - R	2.16	0.033	0.768
Pars triangularis - R	1.99	0.049	0.776
Post superior temporal gyrus - R	2.27	0.025	0.745
Ant inferior temporal gyrus - L	-2.16	0.032	0.768
Inferior temporal gyrus - R	2.30	0.023	0.745
Medial prefrontal cortex	2.29	0.023	0.745
Precuneus	2.88	0.005	0.371
Lingual gyrus - L	2.30	0.023	0.745
Caudate - L	2.08	0.039	0.768
Clustering Coefficient			
Precentral gyrus - L	-2.04	0.043	0.768
Post inferior temporal gyrus - L	3.56	0.001	0.079
Posterior cingulate	4.71	0.000	0.002*
Parietal operculum - R	-2.27	0.025	0.745

* Denotes significance

Supplementary Table 21: Functional graph network measures correlated with CBCL total problems scale based on a robust linear regression. Data showing PME-network interaction terms with p-values < 0.05. A false discovery rate less than 0.05 was considered significant. Clustering coefficient in the posterior cingulate was significant after correcting for multiple comparisons.

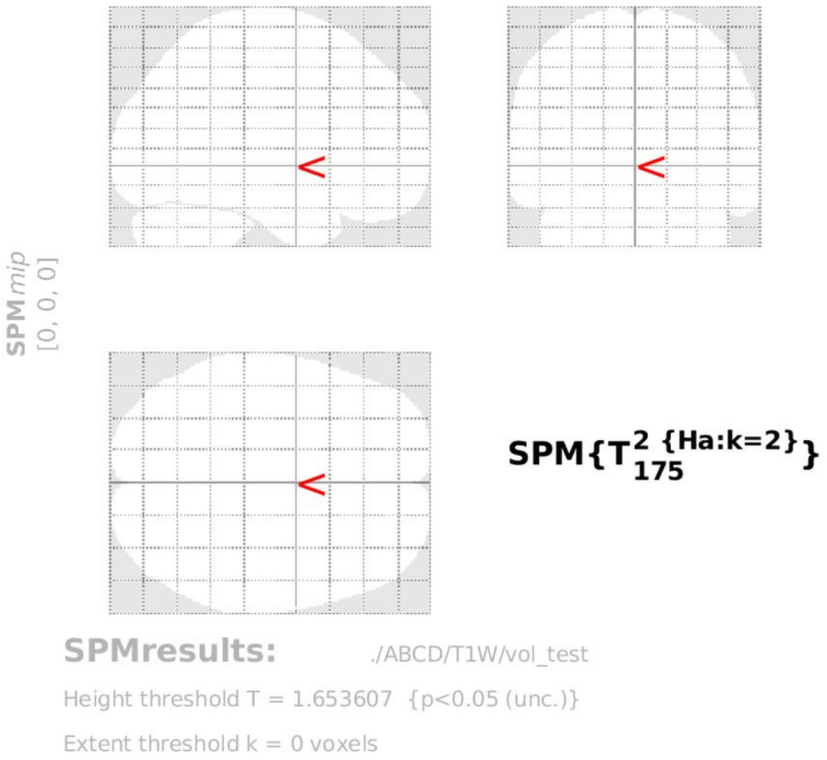

Supplementary Figure 1: Voxel-based morphometry was performed between prenatal marijuana exposed (PME) children and controls. There were no significant differences in any of the voxels between the two groups ($p < 0.05$).

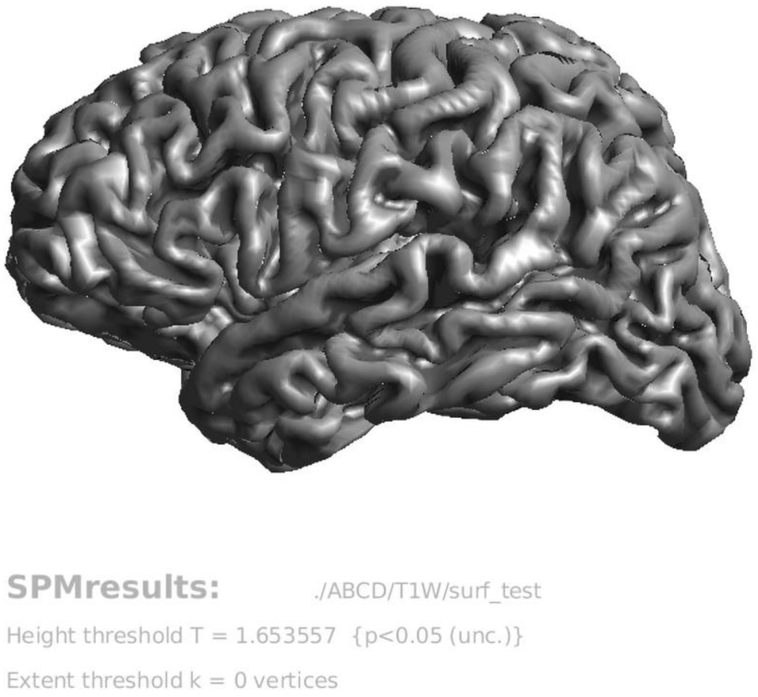

Supplementary Figure 2: Surface-based morphometry was performed between prenatal marijuana exposed (PME) children and controls. There were no significant differences in any of the vertices between the two groups ($p < 0.05$).

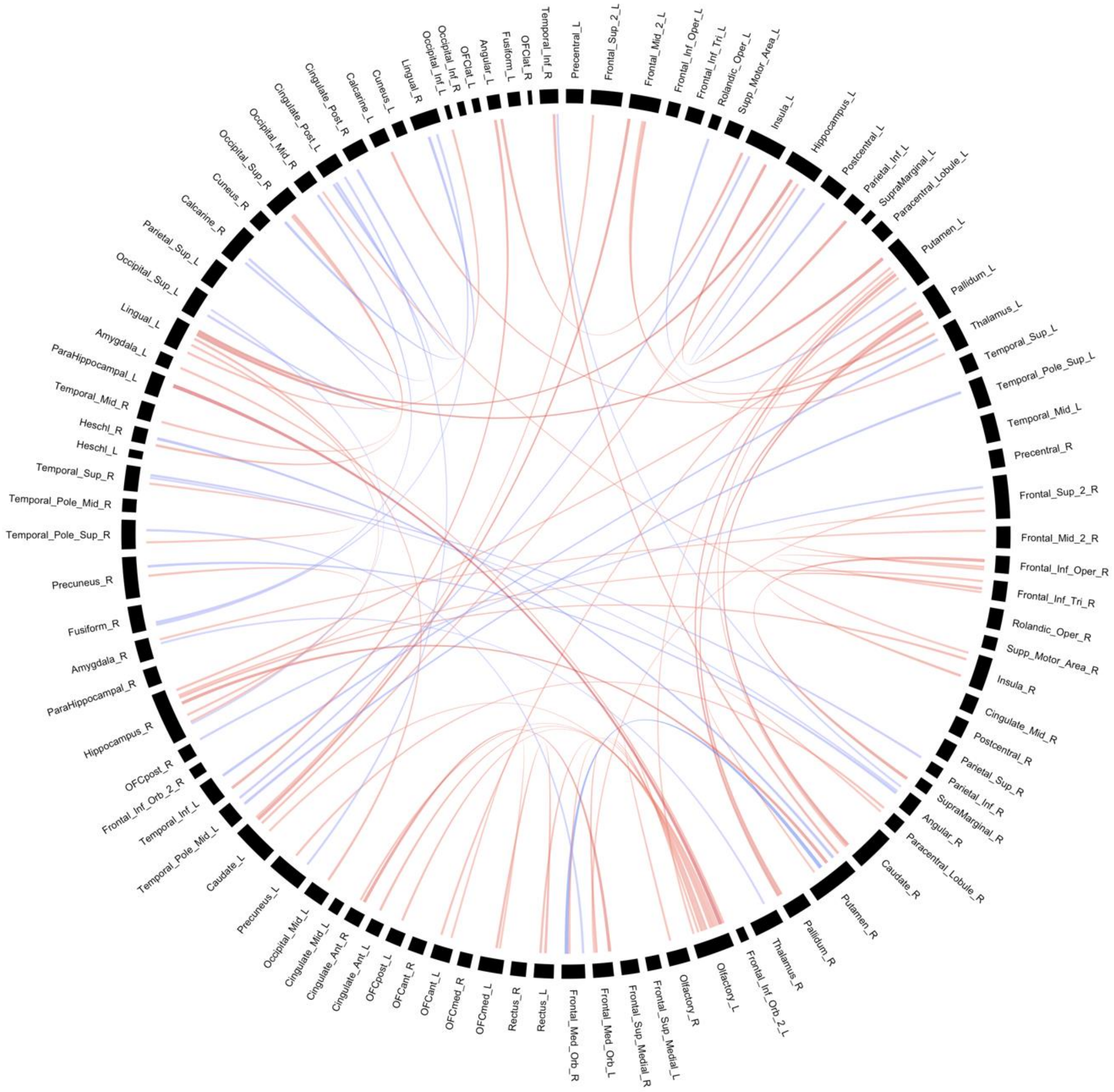

Supplementary Figure 3: Structural connectivity diagram – Showing connectivity values based on 94 regions of interest (ROIs). Values are t-stat scores where red indicates greater connectivity in the PME group while blue indicates greater connectivity in the controls group. Only values that are significant for individual comparisons are shown ($p < 0.05$).

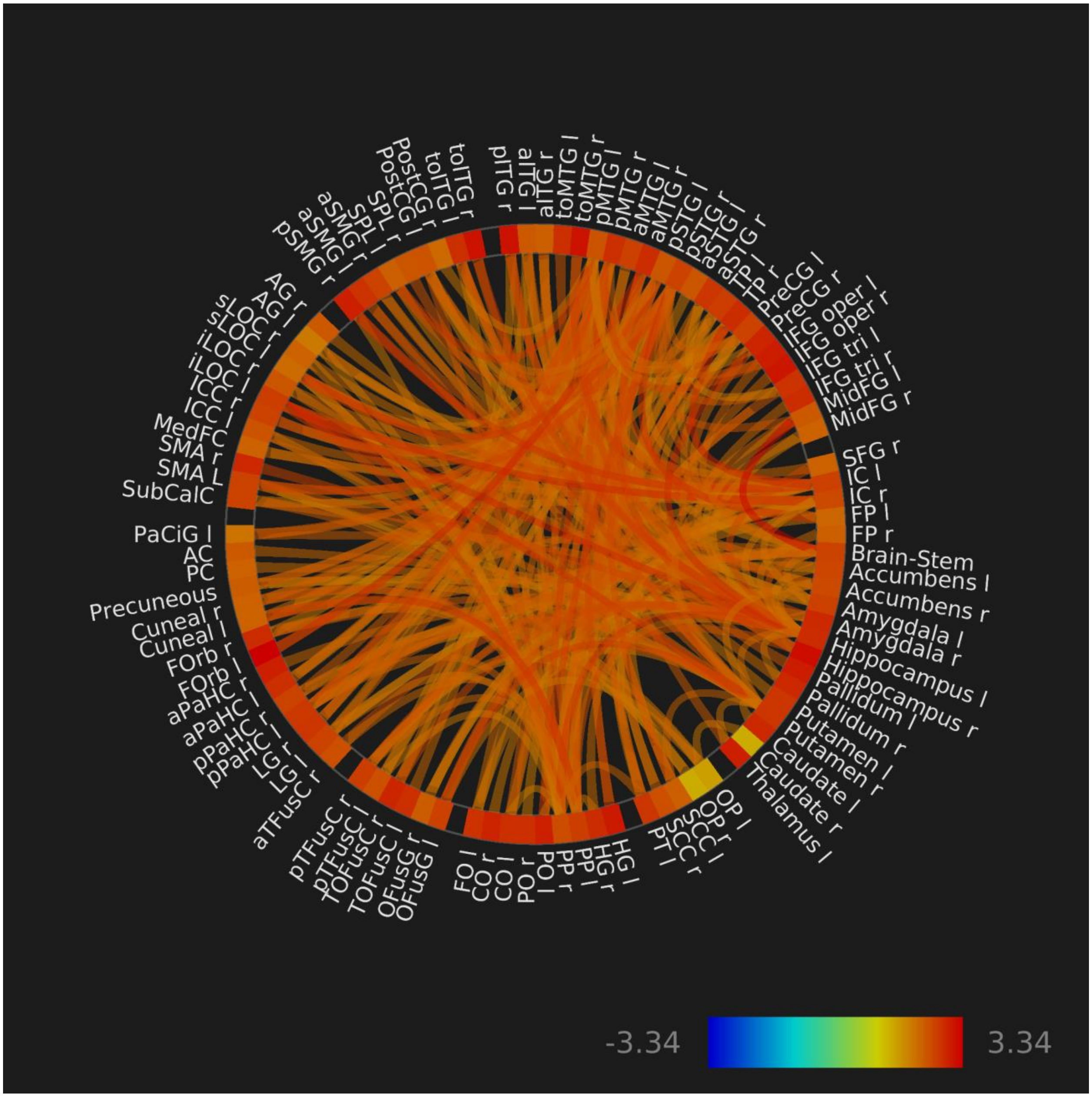

Supplementary Figure 4: Functional connectivity diagram – Showing connectivity values based on 106 regions of interest (ROIs). Values are t-stat scores where red indicates greater connectivity in the PME group while blue indicates greater connectivity in the controls group. Only values that are significant for individual comparisons are shown (P < 0.05).
